# Latest Trends in Industrial Vinegar Production and the Role of Acetic Acid Bacteria: Classification, Metabolism, and Applications—A Comprehensive Review

**DOI:** 10.3390/foods12193705

**Published:** 2023-10-09

**Authors:** Juan J. Román-Camacho, Isidoro García-García, Inés M. Santos-Dueñas, Teresa García-Martínez, Juan C. Mauricio

**Affiliations:** 1 Department of Agricultural Chemistry, Edaphology and Microbiology, Agrifood Campus of International Excellence ceiA3, University of Córdoba, 14014 Córdoba, Spain; b32rocaj@uco.es (J.J.R.-C.); mi2gamam@uco.es (T.G.-M.); mi1gamaj@uco.es (J.C.M.); 2 Department of Inorganic Chemistry and Chemical Engineering, Agrifood Campus of International Excellence ceiA3, Nano Chemistry Institute (IUNAN), University of Córdoba, 14014 Córdoba, Spain; ines.santos@uco.es

**Keywords:** acetic acid bacteria, oxidative metabolism, production system, raw material, taxonomic classification, vinegar

## Abstract

Vinegar is one of the most appreciated fermented foods in European and Asian countries. In industry, its elaboration depends on numerous factors, including the nature of starter culture and raw material, as well as the production system and operational conditions. Furthermore, vinegar is obtained by the action of acetic acid bacteria (AAB) on an alcoholic medium in which ethanol is transformed into acetic acid. Besides the highlighted oxidative metabolism of AAB, their versatility and metabolic adaptability make them a taxonomic group with several biotechnological uses. Due to new and rapid advances in this field, this review attempts to approach the current state of knowledge by firstly discussing fundamental aspects related to industrial vinegar production and then exploring aspects related to AAB: classification, metabolism, and applications. Emphasis has been placed on an exhaustive taxonomic review considering the progressive increase in the number of new AAB species and genera, especially those with recognized biotechnological potential.

## 1. Introduction

Since time immemorial, vinegar has been a fermented foodstuff widely used by mankind as a part of the diet and as a preservative, condiment, and flavoring additive. Because of its bio-healthy properties, vinegar was even traditionally used in ancient medicine [1,2]. Currently, there is a great variety of vinegars around the world, depending on the starter microbial composition, the raw material, and the technical methods used for production [3,4]. From an industrial point of view, the elaboration of vinegar is performed from an alcoholic medium in which a mixed culture of acetic acid bacteria (AAB) is used to carry out a biotransformation process of ethanol into acetic acid, which occurs in specific bioreactors (acetators) [5,6]. Despite the high quality of the final products obtained through traditional systems, including mainly solid-state fermentation and surface culture, their numerous disadvantages, including low efficiency, the slowness of the process, and a lack of control of the operating conditions, have led to the use of the submerged culture system [7,8]. The success of this method, widely implemented in Western and European countries, lies in the high yield and speed of the process under controlled stirring conditions due to the efficiency of mass transfer and continuous vigorous aeration [7,9]. In this sense, the choice of a working mode for the acetators also ensures a suitable environment for the development and activity of AAB, and the control and monitoring of fermentation conditions are some of the fundamental aspects to consider [5,7,10].

On the other hand, vinegar production would not be possible without the activity of the acetic acid bacteria (AAB). These microorganisms, strictly aerobic, can be found in a wide variety of natural and industrial environments. Their versatility and metabolic adaptability make them a taxonomic group of high interest for studying the optimization of obtaining multiple products, with acetic acid as one of the main components, as well as the essential mechanisms that allow them to grow under harsh conditions [11,12,13]. In this sense, the role of their membrane-bound and soluble dehydrogenase system may offer new opportunities in the development of innovative processes based on their capability to carry out the incomplete oxidation of several substrates, including alcohols, sugars, and sugar alcohols, for the production of organic acids [11,14]. Furthermore, the ability of AAB to produce exopolysaccharides is also of great interest for both research and industrial purposes, with some strains considered model organisms for understanding the mechanisms of cellulose synthesis. Moreover, at present, these are the most efficient microorganisms for producing them under controlled conditions [15,16,17]. Finally, the current state of omic technologies and efficient molecular modification methods may be applied to increase the understanding of physiological behavior and the characterization of new strains recovered from these complex media, as well as to exploit the full potential of AAB for producing vinegar and other related bioproducts [6,18,19,20,21,22].

Taking all the above into account, this review aims to provide an update containing some of the recent advances and challenges that have arisen around the main variables controlling industrial vinegar production as well as the characterization of the main microorganisms responsible for the process: AAB. Regarding the latter and considering recent research, which includes a progressive increase in the number of species and the description of new genera, especially those with recognized biotechnological potential, a taxonomic review has been carried out in this work.

## 2. The Historical Context of Vinegar: Origin and Uses

Vinegar has its origin in ancient civilizations. Concretely, the first testimony written on the use of vinegar comes from ancient Babylon, about 5000 years ago, when it was employed as a food preservative. Vinegar was “discovered” fortuitously when undisturbed wine stored in the open air turned spontaneously into vinegar [23]. Due to this phenomenon, known as “wine pitting”, and its sour taste, vinegar has been considered historically as a byproduct with poor commercial interest. However, the numerous applications and benefits of vinegar have been disclosed by mankind throughout history. Hippocrates (460–377 BCE) recommended using vinegar for cleaning ulcerations and for the treatment of sores [24]. Long afterward, in the 10th century, Sung Tse implemented the use of vinegar as a hand-washing agent to prevent infections, which led to an important development in the field of forensic medicine in China [1,25]. In the 18th century, American medical practitioners used vinegar to treat many ailments, including stomachache, high fever, and edema among others [1,24]. Nowadays, vinegar is widely consumed all over the world both directly and indirectly, as it is included in a great variety of dishes including sauces, ketchup, and mayonnaise [1].

**Figure 1:**
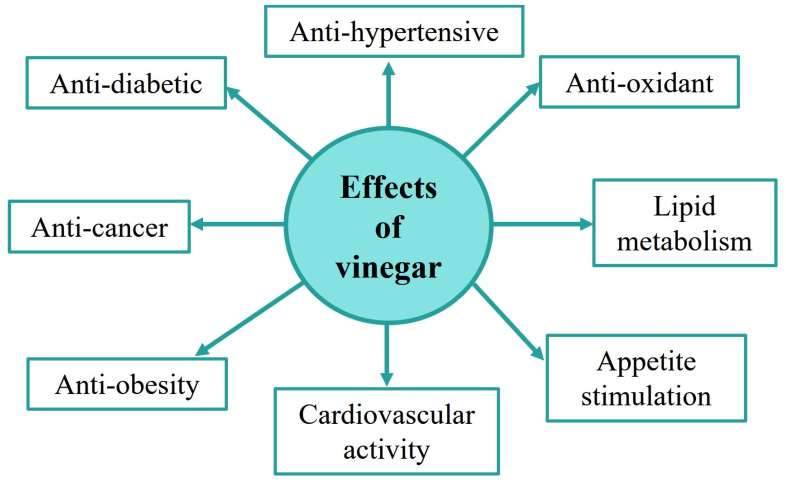
Functional properties and health benefits of vinegar on human metabolism.

Although vinegar has been traditionally used as a flavoring and food preservative, several studies evidence its nutritional potential effects, which can directly affect the health of consumers. This is due, in great part, to the healthy properties that acetic acid (the main constituent of vinegar) can exert on the human liver and gastrointestinal tract [26]. As a consequence, some of the benefits of vinegar may include appetite stimulation, recovery from exhaustion, antioxidant activity, lower lipid content in blood, and the regulation of blood pressure, which, in turn, have an effect on biomarkers for several diseases such as obesity, cancer, diabetes, and hypertension, among others (see Figure 1) [25,26,27,28,29,30,31,32].

## 3. Varieties of Vinegar

There is a great variety of vinegars around the world whose organoleptic properties are conferred by the starter microbial composition, raw material, and technical methods used for their production [3,4]. Vinegar has an alcoholic origin; it usually comes from the processing of vegetables or fruits [25]. In this way, several raw materials can be processed and used as acetification substrates, including wines, spirits, cereal grains (rice wines and malts), and fruit juices, among others. Other raw materials of animal origin can also be used, such as whey or honey [1,33,34]. This section will describe some of the most widely used varieties of vinegar in the world for which the raw materials are specific to particular regions and confer on the final product exceptional organoleptic properties and high quality.

### 3.1. Mediterranean Vinegar: Wine and Balsamic Vinegar

In Mediterranean countries, wine is the most used raw material due to the importance of grapevine cultivation in this region. Wine-producing countries are usually major vinegar-producing countries [19,35]. White and red wines allow for producing a large proportion of total wine vinegars [36]. In Spain, there are three Protected Designations of Origin (PDOs): “Sherry Vinegar”, “Vinegar of Condado de Huelva”, and “Vinegar of Montilla-Moriles”, all of them located in Andalusia [37]. Climate and soil factors of this region permit the growing of native varieties of grapes used for producing high-quality wines, which confer on the final vinegar exceptional organoleptic properties [4]. The aging process by the method known as “Criaderas y Soleras” is one of the singularities of these vinegars, which further enhances their uniqueness. In northern Italy, traditional balsamic vinegar has two PDOs: “L’Aceto Balsamico Tradizionale di Modena” and “L’Aceto Balsamico Tradizionale di Reggio Emilia” [38]. From the native varieties of grapes grown in this region near Modena, a must is obtained that is subsequently cooked. The cooked must is then subjected to simultaneous and spontaneous alcoholic and acetic acid fermentation, followed by a prolonged aging period of at least 12 years using barrels of different types of wood and sizes [38,39].

### 3.2. Spirit Vinegar

Spirit vinegar, also known as white vinegar, is obtained by the acetic acid fermentation of an alcohol medium produced from a previous alcoholic fermentation of an agricultural product containing fermentable sugars [40]. Spirit vinegar can reach the highest acidity levels [15–20% (*w*/*v*)], and although from a sensory point of view its organoleptic profile is usually very poor, in quantitative terms, it is one of the most produced vinegars worldwide, mainly in Great Britain, Germany, and the eastern USA [25,41]. For these reasons, spirit vinegar is used in studies that aim to achieve a high-yield acetification profile. Some of its main applications are as a cleaning product, seasoning, and food preservative [25].

### 3.3. Cereal Vinegar

Traditional cereal vinegar has a long history, with thousands of years of development and improvement that bind it to the Asian continent [42,43]. These vinegars differ according to several factors, such as the type of cereal (such as rice, sorghum, corn, barley and wheat) used as raw material, the microbial composition of starter cultures, elaboration procedures, and aging periods [42]. Among them, rice vinegar, obtained by the acetic acid fermentation of rice wine, “sake”, is popular in Asian countries. In Japan, vinegars are classified into polished rice vinegar (“Komesu”), unpolished rice vinegar (“Kurosu”), sake-less vinegar (“Kasuzu”), and other grain vinegars [44]. In China, different starchy substrates from each region are used for making some of the most famous Chinese vinegars, including Shanxi aged vinegar, Zhenjiang aromatic vinegar, Sichuan bran vinegar, and Fujian *Monascus* vinegar [45,46,47,48]. The particularities of processing the cereal vinegar include the use of solid-state fermentation (SSF), in which the starter culture is previously treated to allow the present dominant microbiota to carry out the saccharification and subsequent alcohol fermentation of the grains just before the acetic acid fermentation, thus producing a high-quality final product that is usually slightly milder and sweeter than Western vinegars [45,49].

### 3.4. Fruit Vinegar

Fruit vinegar is usually elaborated as an alternative for the exploitation of existing fruit surpluses, thus reducing the economic and environmental impact produced by the fruit industry [2]. Although Asian countries were the first to become interested in fruits as raw materials (other than grapes), their use and study in other parts of the world have been increasing in the last two decades [2]. The acidic nature of fruit vinegar and the high sensory impact of acetic acid on its organoleptic properties allow almost any type of fruit to be used for its elaboration. Among them, well-known cider vinegars are elaborated using apple juice through double alcoholic and acetic acid fermentation, especially in the United Kingdom, the United States, and Switzerland [50]. Many other fruits have been explored for the elaboration of vinegars, such as berry, persimmon, strawberry, pineapple, cherry, orange, mango, banana, and tomato, in the last few years [2]. Raw material processing is essential for the extraction of juice; crushing or pressing fruits are usually the most employed methods. Then, both the traditional surface culture and submerged culture can be employed as fermentation systems [2,8,27,50]. Depending on the fruit used, the final product will have a different nutritional composition; in the case of cider vinegar, the high polyphenol content of apples is responsible for its exclusive organoleptic properties, including a high astringency and viscosity as well as numerous health benefits [25,27,50,51].

## 4. Systems of Vinegar Production

Vinegar elaboration can be carried out by either solid-state fermentation (SSF) or liquid fermentation, with the latter including numerous techniques implemented in Western and European countries, mainly surface and submerged cultures [5,7]. The submerged culture is one of the main systems used to produce vinegar on an industrial scale [7,8,9,52].

### 4.1. Traditional Systems: Solid-State Fermentation and Surface Culture

SSF consists of a series of traditional techniques in which the microbiota responsible for the fermentation grow on substrates in the absence of free water [7]. These systems are often used in Asian countries to elaborate vinegar from grains (cereals) and to obtain high-quality vinegar after a period of aging [53,54]. SSF includes three main biological processes: (1) the liquefaction and saccharification of raw material starch, (2) alcohol fermentation, and (3) acetic acid fermentation. However, this method may be slower and less efficient than other techniques [7].

Among these traditional systems, the Orléans, Luxembourgish, and Schützenbach methods are the most used [4,55,56]. The Orléans or French method is the main surface culture system; it consists of an old procedure based on the use of wooden barrels to elaborate vinegar. The substrate used consists of a mixed culture of wine and vinegar, while acetic acid bacteria (AAB) are located on the surface, forming a biofilm known as “the mother of vinegar” [4,57]. During the production process, volumes of vinegar are harvested from the barrels every 8 to 10 days and replaced by wine. The acetification and aging processes occur simultaneously to obtain a high-quality product, but this method is too slow and involves high production costs [56,58]. The Luxembourgian and Schützenbach or German methods implement an immobilization system of AAB using supports made from wood shavings. These systems allow for increasing the contact surface between AAB and the acetification substrate, thus improving the oxygenation of the medium and the acetification yield [59]. Despite the high quality of vinegars obtained by these methods, they show some disadvantages, including the difficulties of controlling the system’s operational variables (such as temperature, oxygen supply, and volatile compounds) and ethanol losses through evaporation [4,56]. In addition, the bioprocess is normally slowed down, and the system does not allow for obtaining final products with acetic acid concentrations higher than 8–10% (*w*/*v*) [56,59].

### 4.2. Submerged Culture System

To industrialize vinegar production, the submerged culture was developed. Through this system, a submerged fermentation process takes place by which the ethanol content of the raw materials (such as spirits, wines, or fruit juice) is oxidized to acetic acid by AAB under controlled stirring conditions [7,8]. This biotransformation is carried out in short periods (24–48 h) and allows us to obtain high-acidity final products. This is mainly possible because of the efficiency of mass transfer and continuous vigorous aeration throughout the process [5,60]. Some aspects that contribute to the high efficiency of this method are as follows.

#### 4.2.1. The Bioreactor: Acetator Frings

The current success of vinegar-making industries is undoubtedly because of the use of the acetator developed by Hromatka and Ebner [61] and marketed by Heinrich Frings GmbH and Co., Bonn, Germany. These bioreactors have stainless steel tanks that can work with different volumes, from a pilot scale (8–10 L) to an industrial scale volume (20,000–100,000 L) [59]. They are equipped with coils as heat exchangers to maintain a constant temperature of 30–31 °C and an efficient volatile recovery system using exhaust gas condensers and scrubbers; as a result, the losses of volatile compounds because of stripping are considerably minimized [5,7,62]. Undoubtedly, the aeration system of these bioreactors confers on them a great part of their success; it consists of a turbine system that sucks air from the outside and releases it inside, resulting in very fine air bubbles, thus generating a homogeneous mixture with the culture medium [5,7,10,63]. Through this system, a higher oxygenation efficiency is reached, thus obtaining higher acetification yields than when using traditional methods.

#### 4.2.2. Operating Modes

**Figure 2:**
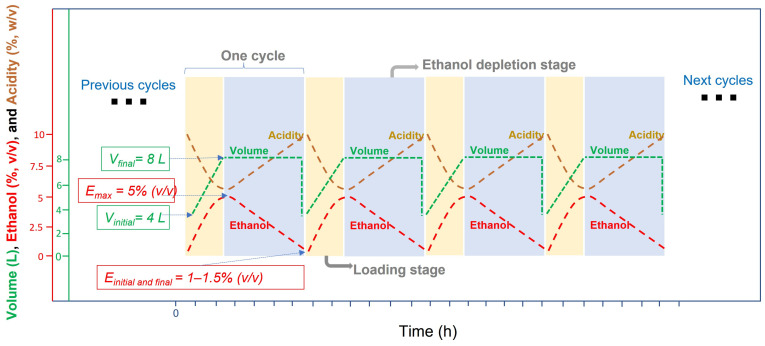
Submerged culture for vinegar production working in a semi-continuous mode. Each cycle of acetification starts by loading the tank to its working volume (8 L) without exceeding a preset ethanol concentration [5% (*v/v*)]. When ethanol concentration is depleted to 1.0–1.5% (*v/v*), 50% of the reactor content (4 L) is unloaded. This system is maintained for the following production cycles.

Another fundamental aspect lies in the operating modes used for these bioreactors, which mainly work in batch, semi-continuous, and continuous ways. Although this choice depends on the specific purpose, which may comprise many factors, in general, a suitable environment for the development and activity of acetic acid bacteria must be ensured [5,7]. According to several authors, AAB may show high sensitivity to different variables, including ethanol concentration, acetic acid concentration, the total strength of the medium (sum of the previous two), temperature, and available dissolved oxygen [64,65,66,67,68]. In this sense, numerous studies have demonstrated that using a continuous mode, a maximum acidity level of 8–10% (*w/v*) can be achieved because a higher concentration or even a low ethanol content can affect the specific growth rate of AAB [7]. On the other hand, the batch mode normally implies lower productivity and additional difficulty in the preparation and maintenance of starter cultures for each cycle [5]. Moreover, the semi-continuous working mode has been mainly imposed for the industrial production of vinegar [5,7,62,66]. In this method, each cycle is started by a loading phase that replenishes the reactor with a fresh medium to the working volume without exceeding a preset ethanol concentration. Then, an exhausting stage occurs, depleting the ethanol in the culture broth to a preset extent. Finally, a fraction of the volume content in the reactor is partially unloaded and the remaining volume is used as inoculum for the next cycle; Figure 2 shows an example of what might be a typical sequence of work in this process [19,52,67]. Working in this way, the operational variables are the initial concentration of ethanol in the culture medium, the concentration to which ethanol must be depleted for a cycle to be finished, the volume of the broth that is then unloaded, and the rate at which the bioreactor is loaded with fresh medium [64,65,67]. Because AAB are highly sensitive to both ethanol and acetic acid, cell concentration and viability can be strongly affected by fermentation conditions [5,60]. Therefore, an appropriate selection of the values of operational variables is essential for maintaining suitable ranges of both substrate and product concentrations, and in this way, the natural self-selection of the best-adapted AAB to the specific working medium is carried out [6,67]. This system also allows for obtaining high-strength vinegars that may reach high acidity levels [up to 15% (*w/v*)]. With the high demand for these products, it may be necessary to use dual-stage high-strength processes in which two fermentation tanks are operated in a synchronous mode, thus even achieving acetic acid concentrations above 20% (*w/v*) [9]. In this way, the stressful environment to which AAB are subjected detracts from the overall acetification rate [5].

#### 4.2.3. Automation Systems

**Figure 3:**
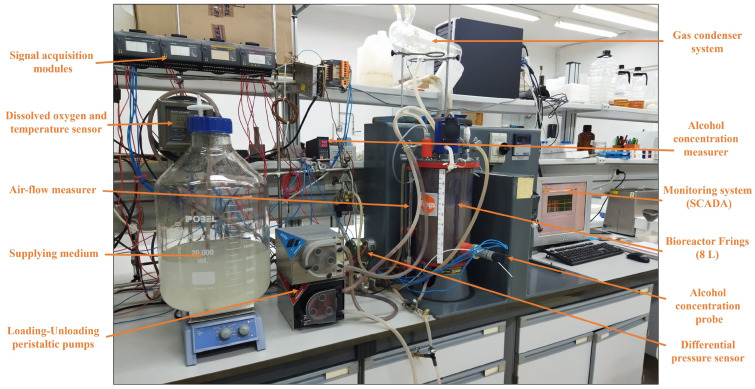
Pilot plant, on a laboratory scale, equipped with a Frings Acetator (8 L) working in a semi-continuous mode and a SCADA automation system that controls the main variables of the system. Biochemical Engineering Laboratory, Chemical Engineering Section, University of Córdoba, Spain.

Each operating mode, particularly the semi-continuous mode, require control and monitoring because the fermentation conditions may induce variations in the development of cycles, even under identical conditions [5,69]. For this, the use of a monitoring system is necessary to obtain a constant recording of data of the main variables to be measured, including the volume of the medium, the concentration of ethanol and dissolved oxygen in it, and temperature [10,66]. Supervisory Control and Data Acquisition (SCADA) consists of monitoring software that provides a set of instructions for specific sensors controlled by signal acquisition modules [5]. This scheduling system also allows for sampling at critical moments of the cycle, such as at specific points in the loading and unloading periods, using different measuring devices including probes and transducers equipped with diverse sensors that continuously monitor and register all the values of each aforementioned main variable [67]. As an example, Figure 3 shows a pilot plant on a laboratory scale, consisting of a Frings acetator (8 L) working in a semi-continuous mode and equipped with a SCADA automation system that allows for the control of the main system variables.

## 5. General Characteristics of Acetic Acid Bacteria

**Figure 4:**
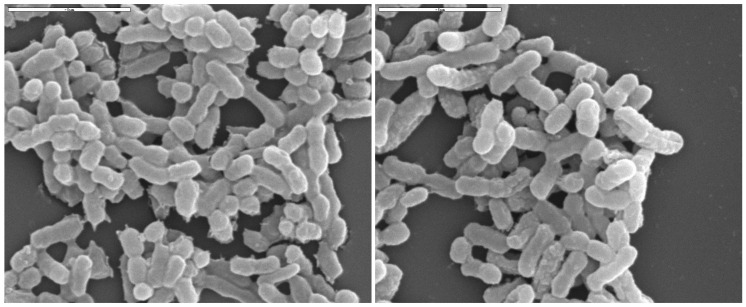
Images taken from a sample (two different planes) of vinegar, which is normally comprised of a complex microbiota of acetic acid bacteria, working inside the bioreactor used (Frings, 8 L) by scanning electron microscopy (SEM).

The vinegar elaboration would not be possible without the activity of the acetic acid bacteria (AAB). These bacteria are Gram-negative or Gram-variable and their metabolism is strictly aerobic, using molecular oxygen (O_2_) as the last electron acceptor. Despite this, some strains of *Acetobacter* and *Gluconobacter* may tolerate low dissolved oxygen concentrations, for example, throughout the alcoholic fermentation in winemaking, when they may potentially be reactivated during the wine clarification [4,12]. AAB are catalase-positive and oxidase-negative, their optimum growing temperature usually ranges between 25 and 30 °C, and their optimum growing pH is between 5 and 6.5, although many AAB do not present difficulties when growing at much lower pH levels, such as between 3 and 4 [13,70,71]. The tolerance to low pH depends on parameters such as ethanol and acetic acid concentrations and oxygen availability [72]. Regarding their shape, most AAB are ellipsoidal or cylindrical, their size usually ranges between 0.4 and 1 μm wide and 0.8 and 4.5 μm long, and they can be observed under the microscope alone, in pairs, or as aggregates and chains (see Figure 4).

These microorganisms constitute a very heterogeneous bacterial group whose cells are normally mobile with peritrichous or polar flagellation. In nature, AAB are found on substrates containing sugars and/or alcohols such as fruit juice, wine, cider, beer, and vinegar [4,11]. On them, sugars and alcohols are incompletely oxidized, thus producing organic acids such as acetic acid from ethanol, mainly performed by the genera *Acetobacter* and *Komagataeibacter*, or gluconic acid from glucose, carried out by the genus *Gluconobacter* [41,73,74]. The ability of AAB to metabolize different carbon sources, such as alcohols and sugars, into organic acids is of great interest to the biotechnological industry; however, vinegar production is still the most extensively used industrial application.

## 6. Current Taxonomy of Acetic Acid Bacteria

Acetic acid bacteria are classified in the Acetobacteraceae family, which is included in the order Rhodospirillales of the class Alphaproteobacteria. Acetobacteraceae consists of two groups: an acetous group and an acidophilic group, based on ecological and phylogenic studies [75,76]. The former includes acetic acid bacteria, which share different a set of general features (see previous section) and include a wide diversity of genera: *Acetobacter*, *Asaia*, *Gluconacetobacter*, *Gluconobacter*, *Granulibacter*, and *Komagataeibacter* among others. The members of the acidophilic group have natures and origins that are physiologically and biochemically heterogeneous, and they include other acidophilic and neutrophilic genera like *Acidiphilum* and *Roseomonas,* among many others [76]. Recent research suggests that Acetobacteraceae family members are a potential source of many undiscovered bacterial metabolites that deserve further experimental exploration [75].

Regarding the acetic acid bacteria (AAB) group, *Acetobacter* was the first proposed genus [77]. In the 1960s, the taxonomy of AAB was significantly influenced by several studies based on the chemotaxonomy of the G + C content of DNA, quinone systems, cellular fatty acid composition, and DNA-DNA hybridization [78]. Throughout time, four main genera of AAB (*Acetobacter*, *Gluconobacter*, *Gluconacetobacter*, and *Komagataeibacter*) were established based on their membrane-bound dehydrogenases, which define their ethanol oxidation capabilities, and the type of respiratory coenzyme chain they contained [79,80]. Through the development of polyphasic classification techniques that integrate the analysis of several phenotypic, chemotactic, and genotypic data, new genera and species have been continuously reported [81]. Further, data from phylogenetic analysis based on 16S ribosomal RNA (rRNA) gene sequences have had a profound impact on the systematics of AAB, as well as the rest of the genera of the Acetobacteraceae family [76].

The activity of AAB is one of the main factors for the elaboration of different types of vinegar [4,6,7]. Species of *Acetobacter* and *Komagataeibacter* are usually among those mainly responsible for acetification because of their high oxidative abilities, although other minor fractions of microorganisms (*Gluconacetobacter*, *Gluconobacter*, among others) might coexist with the best-adapted ones [7,8,21]. *Acetobacter* spp. are widely found in wine, cereal, and balsamic vinegar, elaborated by traditional methods and early stages of those produced by submerged culture or low-acid vinegar because they may be damaged at acidity levels above 8–10% (*w/v*) [7,39]; *Acetobacter pasteurianus* is usually one of the most widely found species [53,73]. *Komagataeibacter* spp. (mainly relocated from *Gluconacetobacter*), which can resist acidity levels of up to 15–20% (*w/v*), are predominant in submerged cultures including spirit vinegar, late stages of the production of several wine and fruit vinegars, and even traditional vinegars, because of their tolerance to low acidity levels [8,41,63]; *Komagataeibacter europaeus* has been described as one of the main AAB for the industrial production of vinegar [19,57,82].

Currently, up to 47 genera and 207 species belonging to the Acetobacteraceae family have been identified according to Hördt et al. [83] and the List of Prokaryotic Names with Standing in Nomenclature (LPSN) database (Parte et al. 2020) [84]. Of them, 20 genera and 108 species currently belong to AAB, as can be seen in Table 1, which recompiles the most updated classification to date.

## 7. Metabolism of Acetic Acid Bacteria

The molecular and biochemical aspects that define the metabolism of acetic acid bacteria are increasingly becoming the target of much research. In this section, a general and updated overview of the main AAB metabolic pathways, especially those related to carbon source assimilation including alcohols, sugars, and sugar alcohols for the production of organic acids, has been provided. It is worth noting that many other related metabolic pathways, partially or completely unknown, are presently being studied by several authors [6,154,155,156,157,158].

### 7.1. Biotransformation of Ethanol to Acetic Acid

The overall oxidative biological reaction that defines the biotransformation of ethanol into acetic acid can be represented as follows:

(1) $$C_{2}H_{5}OH+O_{2}\to {CH}_{3}COOH+H_{2}O\;\Delta H{}^{\circ}=-520\;KJ/mol$$

**Figure 5:**
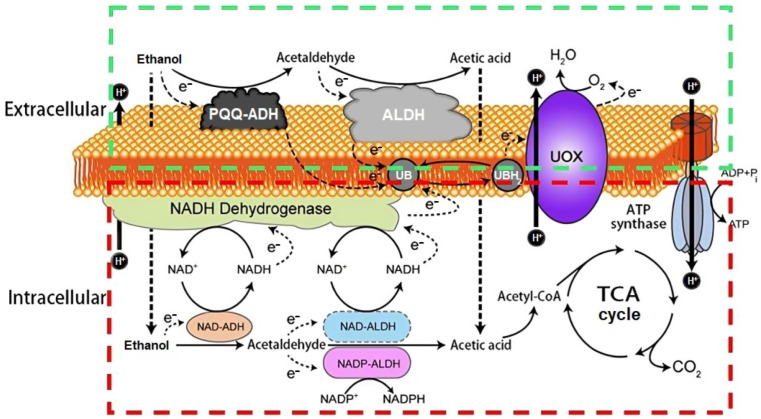
Incomplete oxidation reaction of ethanol into acetic acid both (1) at the cell membrane level (green box): PQQ-ADH, PQQ-dependent alcohol dehydrogenase; ALDH, membrane-bound aldehyde dehydrogenase; UB, ubiquinone; UBH_2_, ubiquinol; UOX, ubiquinol oxidase; and (2) the cytoplasm level (red box): NAD-ADH, NAD-dependent alcohol dehydrogenase; NAD-ALDH, NAD-dependent aldehyde dehydrogenase; NADP-ALDH, NADP-dependent aldehyde dehydrogenase. ATP, energy; TCA cycle, Tricarboxylic Acid Cycle. Adapted by the authors from He et al. [14].

**Figure 6:**
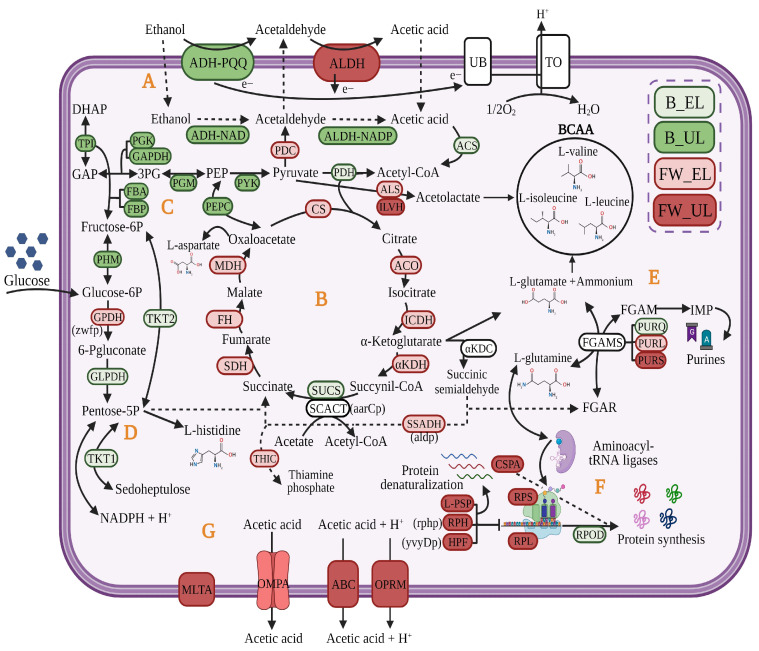
Molecular strategy of *K. europaeus* proposed to prevail throughout acetification process. The colors of the proteins represent the phase in which the protein had the highest quantification value. Fine wine (FW), beer (B), end of loading (EL), just before unloading (UL). A, oxidation of ethanol into acetic acid; B, TCA cycle; C, glycolysis; D, pentose phosphate pathway; E, amino acids and purines formation from biosynthetic precursors; F, regulation of protein synthesis; G, membrane mechanisms for acetic acid release. GAP, glyceraldehyde 3-phosphate; DHAP, dihydroxyacetone phosphate; 3PG, 3-phosphoglycerate; PEP, phosphoenol-pyruvate; FGAR, formylglycinamide ribonucleotide; FGAM, formylglycinamidine ribonucleotide; IMP, inosine monophosphate. Adapted by the authors from Román-Camacho et al. [6].

AAB are chemoorganotrophs microorganisms that use ethanol from a medium of alcoholic origin as a carbon source. The genera *Acetobacter* and *Komagataeibacter* usually show a higher ethanol preference, although other AAB groups may show a preference for other carbon sources [7,41]. This biotransformation consists of an incomplete oxidation reaction of two steps. First, alcohol dehydrogenase (ADH) binds to pyrroloquinoline quinone (PQQ) to oxidize the ethanol into acetaldehyde. Next, acetaldehyde is oxidized to acetic acid by membrane-bound aldehyde dehydrogenase (ALDH); both enzymes are located on the periplasmic side of the inner cell membrane [159,160]. Oxidized nicotinamide adenine dinucleotide (NAD^+^) and nicotinamide adenine dinucleotide phosphate (NADP^+^), both located in the cytoplasm, may be used as coenzymes by NAD-ADH, NAD-ALDH, and NADP-ALDH [156,161]. The inner acetic acid can be completely oxidized by the acetyl-CoA synthase, which leads to the input of acetyl-CoA into the TCA cycle, and in this case, to CO_2_ and H_2_O providing energy (ATP) and detoxifying the cell [14]. Other organic acids such as lactic, pyruvic, malic, succinic, citric, and fumaric acids may be similarly metabolized [73]. Because of the strictly aerobic metabolism of AAB, the ADH-PQQ and ALDH complexes are closely linked to the respiratory chain, which transfers reducing equivalents from donor substrates to ubiquinone (UB). Then, electrons from the reduced UB, named ubiquinol (UBH_2_), are transferred to the final electron acceptor, oxygen (O_2_), by terminal ubiquinol oxidases (UOXs), producing H_2_O (see Figure 5) [14,161]. Some processes related to this central oxidative metabolism may include pathways that aim to obtain biosynthetic precursors of amino acids and nucleic acids, among others, in order to replenish cell material losses throughout the early stages of the acetification process. The TCA cycle also plays a crucial role in the assimilation of internal acetic acid; among the involved enzymes, succinyl-CoA: acetate CoA transferase (SCACT), encoded by *aarC*, New Orleans, LA, USA, is able to produce acetyl-CoA from inner acetate, being of significant importance in the tolerance to acetic acid [162,163]. Furthermore, membrane mechanisms dependent on proton motive force may be triggered for acetic acid release and cell detoxification at the final moments of the process; here, the importance of outer membrane proteins (OMPA) and efflux pumps (OPRM and ABC-transporters) in the control of the cellular output of acetic acid, as well as MLTA, participating in the maintenance of the peptidoglycan layer under these conditions, are highlighted; see Figure 6 [6].

The ADH complex of most AAB is composed of three subunits, although it may contain two subunits in some species [164]. Subunit I (72–78 kDa), encoded by the gene *adhA*, is a catalytic component containing a PQQ and a heme C moiety. Subunit II (44–45 kDa), encoded by the gene *adhB*, is a membrane-anchoring and ubiquinone-reducing component possessing three heme C moieties; both subunits participate in the intramolecular electron transport to the terminal UB. Subunit III (20 kDa), encoded by the gen *adhS*, which has no prosthetic group, facilitates the association of subunits I and II to the membrane and acts as a molecular chaperone for the folding and/or maturation of subunit I [73,161]. Several authors have related high ADH stability and activity with a high tolerance and production of acetic acid, mainly in species from the current genus *Komagataeibacter* [41,82]. The ALDH complex is composed of two or three subunits depending on the AAB species, and it acts as an operon. Although its optimum pH ranges between 4 and 5, the oxidation of acetaldehyde to acetate may be catalyzed at lower pH values. ALDH is highly sensitive to low oxygen concentrations and the presence of ethanol in the medium [73].

### 7.2. Carbohydrates Oxidation

**Figure 7:**
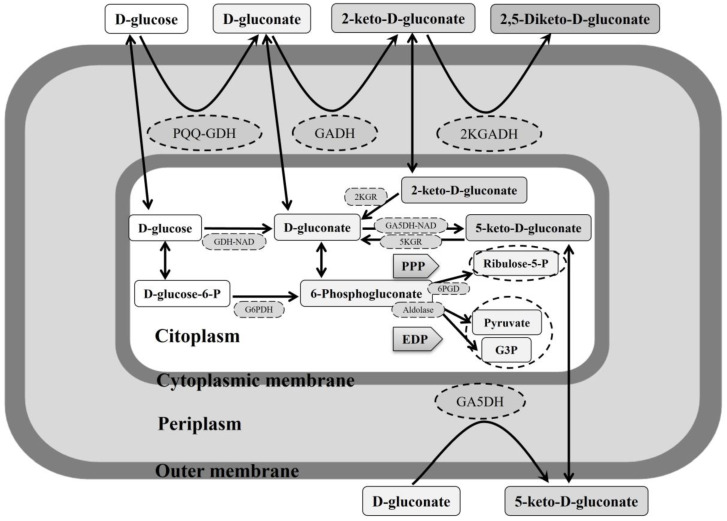
Glucose metabolism in *Gluconobacter*. Cell membrane enzymes: PQQ-GDH, PQQ-dependent D-glucose dehydrogenase; GADH, FAD-dependent D-gluconate 2-dehydrogenase; 2KGADH, FAD-dependent 2-keto-D-gluconate dehydrogenase; GA5DH, PQQ-dependent D-gluconate 5-dehydrogenase. Cytoplasm enzymes: GDH-NAD, NADP-dependent D-glucose dehydrogenase; GA5DH-NAD, NADP-dependent D-gluconate 5-dehydrogenase; 2KGR, 2-keto-D-gluconate reductase; 5KGR, 5-keto-D-gluconate reductase; G6PDH, glucose-6-phosphate dehydrogenase; 6PGD, 6-phosphogluconate dehydrogenase. Compounds: G3P, glyceraldehyde 3-phosphate. Pathways: PPP, pentose phosphate pathway; EDP, Entner Doudoroff pathway. Adapted by the authors from García-García et al. [166].

AAB can metabolize different carbohydrates as carbon sources, mainly glucose, but also arabinose, fructose, galactose, mannose, ribose, sorbose, and xylose [73]. Most AAB have been characterized by non-functional glycolysis because of the absence of a phosphofructokinase enzyme; therefore, the pentose phosphate pathway (PPP) is the main metabolic route of AAB to oxidize the glucose available in the medium by the catalytic activity of the enzymes glucose-6-P dehydrogenase (G6PDH) and 6-phosphogluconate dehydrogenase (6PGD), providing metabolic precursors such as ribulose-5-phosphate and generating NADPH + H^+^ and energy [165,166,167]. Among AAB, several species from *Gluconobacter* have a glucose preference, and several *Gluconobacter oxydans* strains also exhibit the ability to oxidize glucose to gluconic acid via glucono-δ-lactone, forming D-gluconate. This oxidation reaction occurs in the periplasm using a membrane-bound pyrroloquinoline quinone-dependent glucose dehydrogenase (PQQ-GDH) located on the outer side of the cytoplasmic membrane. D-gluconate can be further oxidized rapidly to ketogluconates such as 2-ketogluconate (2-KGA), 5-ketogluconate (5-KGA), and 2,5-diketogluconic acid (2,5-DKGA), both in the periplasm and cytoplasm, by different oxidizing enzymes; see Figure 7 [166,168,169]. Glucose, gluconic acid, and ketogluconates can be assimilated by these bacteria, thus obtaining biomass and energy and acidifying the medium, possibly as part of their metabolic strategy to prevail over other glucose-like microorganisms [166]. Final products of PPP and the Entner Doudoroff pathway (EDP) may be completely oxidized to CO_2_ and H_2_O by *Acetobacter*, *Gluconacetobacter*, and *Komagataeibacter* spp. using the TCA cycle when the carbon source of the medium is exhausted; this is not the case for *Gluconobacter* spp., which show a non-functional TCA cycle [73,169]. Recent studies have proposed a molecular strategy in which *K. europaeus*, as the predominant species of a complex microbiota involved in the submerged production of vinegar from raw materials with high sugar content, might assimilate, firstly and before the ethanol, the glucose in the medium, draining biosynthetic precursors directly by using enzymes of PPP and the glycolysis to prevail over other species that exhibit high glucose preference [6]; see Figure 6.

AAB also exhibit the ability to oxidize several sugar alcohols such as glycerol, D-mannitol, and D-sorbitol, among others, with the use of glycerol as a carbon source being especially remarkable in winemaking, producing dihydroxyacetone (DHA) through the activity of some oxidizing enzymes, mainly glycerol dehydrogenase, and providing energy (ATP) via gluconeogenesis. Strains from *Acetobacter pasteurianus*, *Gluconobacter oxydans*, and *Komagataeibacter xylinus* are some of the most studied regarding this oxidative pathway [16,73].

## 8. Biotechnological Applications of Acetic Acid Bacteria

AAB are the main microorganisms responsible for vinegar production, but they are also used in different increasingly investigated biotechnological applications. Other foods can be produced as the result of the activity of AAB, as is the case of kombucha—a traditional beverage obtained by fermenting sugary tea with a symbiotic culture of acidophilic yeasts and bacteria including acetic acid bacteria (AAB) and lactic acid bacteria (LAB) immobilized in a microbial cellulose biofilm known as tea fungus [71,170]. First, yeasts transform sugars from tea into organic acids, ethanol, and CO_2_. Then, AAB may synthesize different compounds such as acetic acid (*Acetobacter aceti*, *Acetobacter pasteurianus*), gluconic acid (*Gluconobacter oxydans*), and bacterial cellulose (*Komagataeibacter xylinus*) due to the high biodiversity of AAB. This product is becoming more and more popular because of its probiotic characteristics as a treatment for gastrointestinal disorders and for improving general health and increasing longevity, with the benefits attributed to its acidic composition and high phenolic antioxidant content [71,170,171]. Another product, gluconic acid, is industrially obtained by the oxidation of glucose by several AAB, mainly *Gluconobacter oxydans*. Gluconic acid improves the sensory properties and increases the softness of other food products, including vinegar, and may also be used as an additive and preservative by the food industry. Due to its role in the aromatic profile of foods, gluconic acid has been proposed as a quality parameter of food products [71,172]. Gluconic acid is used in the pharmaceutical industry as gluconates of divalent metals, which function as mineral supplements to treat some diseases [173]. The high oxidative capability of numerous strains of *Gluconobacter* is also exploited to convert sugar alcohols, as in the case of D-sorbitol to L-sorbose—an important intermediate in the industrial production of L-ascorbic acid (vitamin C), an antioxidant often used in the food industry [73].

Among the biotechnological applications of AAB, the production of bacterial cellulose has attracted interest in recent years because of its extreme purity, unlike plant-derived cellulose, thus representing a promising alternative for many industries [71]. Among its multiple applications, bacterial cellulose is employed as a gelling, stabilizing, and thickening agent in foods, heart medicine, pharmacy, and skin repair in wound healing and burn treatments [174,175,176]. *K. xylinus* is the most commonly used species of AAB because of its capability to produce high amounts of bio-cellulose from different carbon and nitrogen sources, and this involves different enzymes such as glucose kinase, phosphoglucomutase, UDP-glucose pyrophosphorylase, and membrane-bound cellulose synthase [17]. Throughout strawberry vinegar fermentation, *Ameyamaea*, *Gluconacetobacter,* and *Komagataeibacter* were found, combining both culture-dependent and culture-independent methodologies, as the dominating bacterial genera in biofilms previously generated during the process [177]. Besides bio-cellulose, AAB may produce other microbial exopolysaccharides, such as levans, dextran, acetan, mannan, and gluconacetan, with important industrial applications [15,71].

Omics technologies have emerged in recent years as an alternative to solve many of the traditional hurdles for the isolation and characterization of acetic acid bacteria, especially those that limit the study of the richness and biodiversity of these microbiota inhabiting such selective media as vinegar. In fact, in the past few years, different omic and meta-omic approaches have been implemented, including (meta)genomics [8,21,49,177], transcriptomics [157,178], (meta)proteomics [6,19,41,158], and metabolomics [43,46,48]. These studies are allowing for the identification and characterization of most of the members of these communities involved in these production processes, describing their behavior both under different operating conditions and media, thus helping to elucidate the key role of the vinegar-making microbiota.

Using innovative shotgun metaproteomics (LC-MS/MS) and metagenomic techniques (16S rRNA amplicon sequencing), Román-Camacho et al. [6,21] approach the difficult problem of identifying and characterizing these complex microbial communities, as well as the influence of the use of various culture media for vinegar production; *Komagataeibacter* members, especially *Komagataeibacter europaeus* strains, were the main representatives. Likewise, these works explain the ability of AAB to adapt to different culture media through metabolic versatility. Shotgun metagenomics, which implements the use of sequencing data to infer potential metabolic functions encoded by the genomes of the community members, was applied to reveal the flavor metabolic network of the microbiota of a cereal vinegar [49].

Another aspect that also receives a lot of attention is related to the study of how AAB resist the aggressive environments in which they are normally found—for example, the high acidity values during the industrial production of vinegar. Using RNA-Seq transcriptomic analyses, Yang et al. [179] studied gene regulation changes to find possible relationships with the acidity of the medium, and Wang et al. [157] determined several mechanisms against high acid stress at different stages of acetic acid fermentation in a *K. europaeus* strain.

Metabolomics is another recent approach that, in the vinegar production area (especially Chinese cereal vinegars), is allowing us to determine different volatile aroma patterns throughout fermentation [43], as well as correlate numerous metabolites (volatile compounds, organic acids, and amino acids) with the main members of these microbiotas [46,48]; in these studies, head-space solid phase microextraction (HS-SPME) coupled with gas chromatography–mass spectrometry (GC–MS) was used for determining volatile compounds, and high-performance liquid chromatography (HPLC) was used for organic acids and amino acids.

## 9. Conclusions and Future Prospects

This review has attempted to summarize the current state of knowledge on vinegar production from the diversity of raw materials and starter cultures to system production and operating conditions used. Special emphasis has been placed on submerged cultures as a suitable method of industrial vinegar production and an updated taxonomic review of the main responsible microorganisms for the process: acetic acid bacteria. Because of the multiple variables that influence vinegar production, it is not easy for the industries to achieve a balance between them that is aimed at the optimization of this process. Considering current research and the evolution of the agri-food market, the improvement of the organoleptic properties of these unique products might be focused on the implementation of new operating conditions, the characterization of new raw materials, and the study of the microbial composition and behavior of microbiota inhabiting vinegar, especially those responsible for its elaboration. In recent years, omics tools have approached these strategies with high throughput without compromising the fitness of microbiota and the quality of the final product. In vinegar, they are allowed to solve many of the traditional hurdles for the isolation and characterization of microorganisms inhabiting these aggressive media. Despite this, the current omic analyses still have numerous limitations, such as the challenge of detecting low-abundant species, high levels of technical and biological noise, and the performance of few biological replicates because of the high cost. There is a pressing need to advance the field of sequencing, mass spectrometry technologies, and bioinformatics, implementing robust techniques for integrating, visualizing, and validating omic data. New challenges for industrial vinegar production might target obtaining innovative varieties of vinegar with sensorial profiles and healthy organoleptic properties; the employment of improved starter cultures from the selection of species or strains with a key role throughout acetification; advancements in obtaining vinegar isolates, their phenotypic characterization, and biotechnological enhancement; and the identification of marker genes, proteins, and metabolites throughout the process. This work might establish the first steps of a path toward the improvement of vinegar processing in the industry.

## Figures and Tables

**Table 1:** Current classification of the acetic acid bacteria group. A total of 20 genera and 108 species are shown. Source: LPSN database (https://www.bacterio.net (accessed on 10 June 2022)).

Genera	Species		References
*Acetobacter*	1	*Acetobacter aceti*	Skerman et al. [77]
	2	*Acetobacter cerevisiae*	Cleenwerck et al. [85]
	3	*Acetobacter cibinongensis*	Lisdiyanti et al. [86]
	4	*Acetobacter conturbans*	Sombolestani et al. [87]
	5	*Acetobacter estunensis*	Lisdiyanti et al. [88]
	6	*Acetobacter fabarum*	Cleenwerck et al. [89]
	7	*Acetobacter fallax*	Sombolestani et al. [87]
	8	*Acetobacter farinalis*	Tanasupawat et al. [90]
	9	*Acetobacter garciniae*	Yukphan et al. [91]
	10	*Acetobacter ghanensis*	Cleenwerck et al. [92]
	11	*Acetobacter indonesiensis*	Lisdiyanti et al. [88]
	12	*Acetobacter lambici*	Spitaels et al. [93]
	13	*Acetobacter lovaniensis*	Lisdiyanti et al. [88]
	14	*Acetobacter malorum*	Cleenwerck et al. [85]
	15	*Acetobacter musti*	Ferrer et al. [94]
	16	*Acetobacter nitrogenifiens*	Dutta and Gachhui [95]
	17	*Acetobacter oeni*	Silva et al. [96]
	18	*Acetobacter okinawensis*	Lino et al. [97]
	19	*Acetobacter orientalis*	Lisdiyanti et al. [86]
	20	*Acetobacter orleanensis*	Lisdiyanti et al. [88]
	21	*Acetobacter oryzoeni*	Baek et al. [98]
	22	*Acetobacter oryzifermentans*	Kim et al. [99]
	23	*Acetobacter papayae*	Lino et al. [97]
	24	*Acetobacter pasteurianus*	Skerman et al. [77]
	25	*Acetobacter persici*	Lino et al. [97]
	26	*Acetobacter pomorum*	Sokollek et al. [100]
	27	*Acetobacter sacchari*	Vu et al. [101]
	28	*Acetobacter senegalensis*	Ndoye et al. [102]
	29	*Acetobacter sicerae*	Li et al. [103]
	30	*Acetobacter suratthaniensis*	Pitiwittayakul et al. [104]
	31	*Acetobacter syzygii*	Lisdiyanti et al. [86]
	32	*Acetobacter thailandicus*	Pitiwittayakul et al. [105]
	33	*Acetobacter tropicalis*	Lisdiyanti et al. [88]
*Acidomonas*	34	*Acidomonas methanolica*	Urakami et al. [106]
*Ameyamaea*	35	*Ameyamaea chiangmaiensis*	Yukphan et al. [107]
*Asaia*	36	*Asaia astilbis*	Suzuki et al. [108]
	37	*Asaia bogorensis*	Yamada et al. [109]
	38	*Asaia krungthepensis*	Yukphan et al. [110]
	39	*Asaia lannensis*	Malimas et al. [111]
	40	*Asaia platycodi*	Suzuki et al. [108]
	41	*Asaia prunellae*	Suzuki et al. [108]
	42	*Asaia siamensis*	Katsura et al. [112]
	43	*Asaia spathodeae*	Kommanee et al. [113]
*Bombella*	44	*Bombella apis*	Yun et al. [114]
	45	*Bombella favorum*	Hilgarth et al. [115]
	46	*Bombella intestini*	Li et al. [116]
	47	*Bombella mellum*	Hilgarth et al. [115]
*Commensalibacter*	48	*Commensalibacter intestini*	Roh et al. [117]
*Endobacter*	49	*Endobacter medicaginis*	Ramírez-Bahena et al. [118]
*Gluconacetobacter*	50	*Gluconacetobacter aggeris*	Nishijima et al. [119]
	51	*Gluconacetobacter asukensis*	Tazato et al. [120]
	52	*Gluconacetobacter azotocaptans*	Fuentes-Ramírez et al. [121]
	53	*Gluconacetobacter diazotrophicus*	Yamada et al. [122]
	54	*Gluconacetobacter dulcium*	Sombolestani et al. [123]
	55	*Gluconacetobacter entanii*	Schüller et al. [124]
	56	*Gluconacetobacter johannae*	Fuentes-Ramírez et al. [121]
	57	*Gluconacetobacter liquefaciens*	Yamada et al. [122]
	58	*Gluconacetobacter sacchari*	Franke et al. [125]
	59	*Gluconacetobacter takamatsuzukensis*	Nishijima et al. [119]
	60	*Gluconacetobacter tumulicola*	Tazato et al. [120]
	61	*Gluconacetobacter tumulisoli*	Nishijima et al. [119]
*Gluconobacter*	62	*Gluconobacter aidae*	Yukphan et al. [126]
	63	*Gluconobacter albidus*	Yukphan et al. [127]
	64	*Gluconobacter cadivus*	Sombolestani et al. [128]
	65	*Gluconobacter cerevisiae*	Spitaels et al. [129]
	66	*Gluconobacter cerinus*	Yamada and Akita [130]
	67	*Gluconobacter frateurii*	Mason and Claus [131]
	68	*Gluconobacter japonicus*	Malimas et al. [132]
	69	*Gluconobacter kanchanaburiensis*	Malimas et al. [133]
	70	*Gluconobacter kondonii*	Malimas et al. [134]
	71	*Gluconobacter morbifer*	Roh et al. [117]
	72	*Gluconobacter oxydans*	De Ley [135]
	73	*Gluconobacter potus*	Sombolestani et al. [128]
	74	*Gluconobacter roseus*	Malimas et al. [136]
	75	*Gluconobacter sphaericus*	Malimas et al. [137]
	76	*Gluconobacter thailandicus*	Tanasupawat et al. [138]
	77	*Gluconobacter vitians*	Sombolestani et al. [126]
	78	*Gluconobacter wancherniae*	Yukphan et al. [139]
*Granulibacter*	79	*Granulibacter bethesdensis*	Greenberg et al. [140]
*Komagataeibacter*	80	*Komagataeibacter diospyri*	Naloka et al. [141]
	81	*Komagataeibacter europaeus*	Yamada et al. [80]
	82	*Komagataeibacter intermedius*	Yamada et al. [80]
	83	*Komagataeibacter kakiaceti*	Yamada [142]
	84	*Komagataeibacter kombuchae*	Yamada et al. [80]
	85	*Komagataeibacter medellinensis*	Yamada [142]
	86	*Komagataeibacter melaceti*	Marič et al. [143]
	87	*Komagataeibacter melomenusus*	Marič et al. [143]
	88	*Komagataeibacter nataicola*	Yamada et al. [80]
	89	*Komagataeibacter oboediens*	Yamada et al. [80]
	90	*Komagataeibacter rhaeticus*	Yamada et al. [80]
	91	*Komagataeibacter saccharivorans*	Yamada et al. [80]
	92	*Komagataeibacter sucrofermentans*	Yamada et al. [80]
	93	*Komagataeibacter swingsii*	Yamada et al. [80]
	94	*Komagataeibacter xylinus*	Yamada et al. [80]
*Kozakia*	95	*Kozakia baliensis*	Lisdiyanti et al. [144]
*Neoasaia*	96	*Neoasaia chiangmaiensis*	Yukphan et al. [145]
*Neokomagataea*	97	*Neokomagataea tanensis*	Yukphan et al. [146]
	98	*Neokomagataea thailandica*	Yukphan et al. [146]
*Nguyenibacter*	99	*Nguyenibacter vanlangensis*	Vu et al. [147]
*Novacetimonas*	100 101	*Novacetimonas cocois* *Novacetimonas hansenii*	Brandao et al. [148] Brandao et al. [148]
	102	*Novacetimonas maltaceti*	Brandao et al. [148]
	103	*Novacetimonas pomaceti*	Brandao et al. [148]
*Saccharibacter*	104	*Saccharibacter floricola*	Jojima et al. [149]
*Swaminathania*	105	*Swaminathania saitolerans*	Loganathan and Nair [150]
*Swingsia*	106	*Swingsia samuiensis*	Malimas et al. [151]
*Tanticharoenia*	107	*Tanticharoenia aidae*	Vu et al. [152]
	108	*Tanticharoenia sakaeratensis*	Yukphan et al. [153]

## Data Availability

The data used to support the findings of this study can be made available by the corresponding author upon request.
